# Contemporaneous varicella and zoster or disseminated zoster? A diagnostic challenge in an HIV-positive child

**DOI:** 10.4102/sajid.v41i1.824

**Published:** 2026-05-25

**Authors:** Wendy Batyi, Tshepile Tlali, Preethi John, Matilda Mphahlele

**Affiliations:** 1 Department of Dermatology, Tambo Memorial Hospital, Gauteng Department of Health, Johannesburg, South Africa; 2 Department of Anatomical Pathology, Faculty of Health Sciences, National Health Laboratory Service (NHLS), Johannesburg, South Africa; 3 School of Pathology, Faculty of Health Sciences, University of the Witwatersrand, Johannesburg, South Africa; 4 Department of Internal Medicine, Division of Dermatology, Faculty of Health Sciences, University of the Witwatersrand, Johannesburg, South Africa

**Keywords:** varicella, zoster, Varicella-Zoster virus, contemporaneous, disseminated

## Abstract

Primary infection with Varicella-Zoster virus causes varicella (chickenpox). Thereafter, following reactivation after a period of latency, it can cause zoster (shingles). Simultaneous occurrence of varicella and zoster is rare, particularly in children.

**Contribution:**

We describe an 11-year-old girl, HIV-positive, but not receiving antiretrovirals, who presented with contemporaneous varicella and zoster, with the possibility of disseminated zoster also considered.

## Introduction

Varicella-Zoster virus (VZV) is a globally distributed, highly infectious herpes virus. It is airborne, with transmission via respiratory droplets or through direct contact with fluid within the blisters it causes. It causes two clinically distinguishable diseases, varicella (chickenpox) and zoster (shingles).^1^ Varicella represents primary infection with VZV. Following an average incubation period of 10–21 days, patients develop erythematous, pruritic lesions that progress from macules to papules to vesicles and finally to crusts. Accompanying systemic symptoms may also occur.^2^

Zoster occurs with reactivation of latent VZV infection. During primary infection, VZV migrates to the dorsal root ganglia or sensory ganglia of cranial nerves, where it remains dormant until reactivation. With reactivation, it moves down to the axon to cause skin lesions in the relevant dermatome. Skin lesions may be preceded by a painful, burning sensation, followed by evolution to vesicles.^3^

Rapid reactivation of VZV resulting in zoster prior to resolution of varicella lesions is very rare in children and has only been reported in a few cases. Most published cases describe zoster in children who have received VZV vaccine.^4,5^ We describe a case of contemporaneous varicella and zoster in an 11-year-old girl living with HIV, with a differential diagnosis of disseminated zoster. She had received antiretrovirals postnatally, but had no previous varicella episode on history, and had not been vaccinated with VZV vaccine. Two similar cases, occurring in immunocompetent children, have been described.^1,6^

## Case report

**FIGURE 1:**
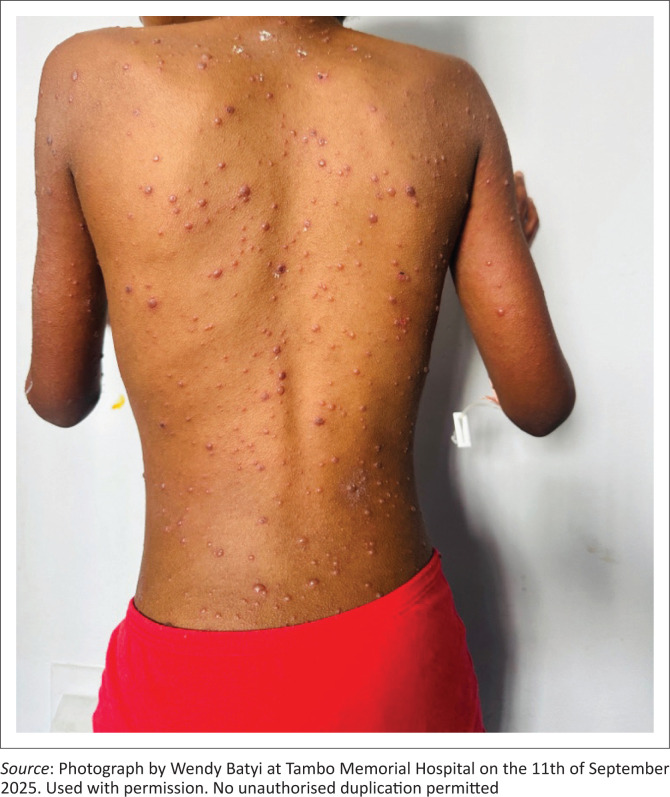
Varicella lesions.

**FIGURE 2:**
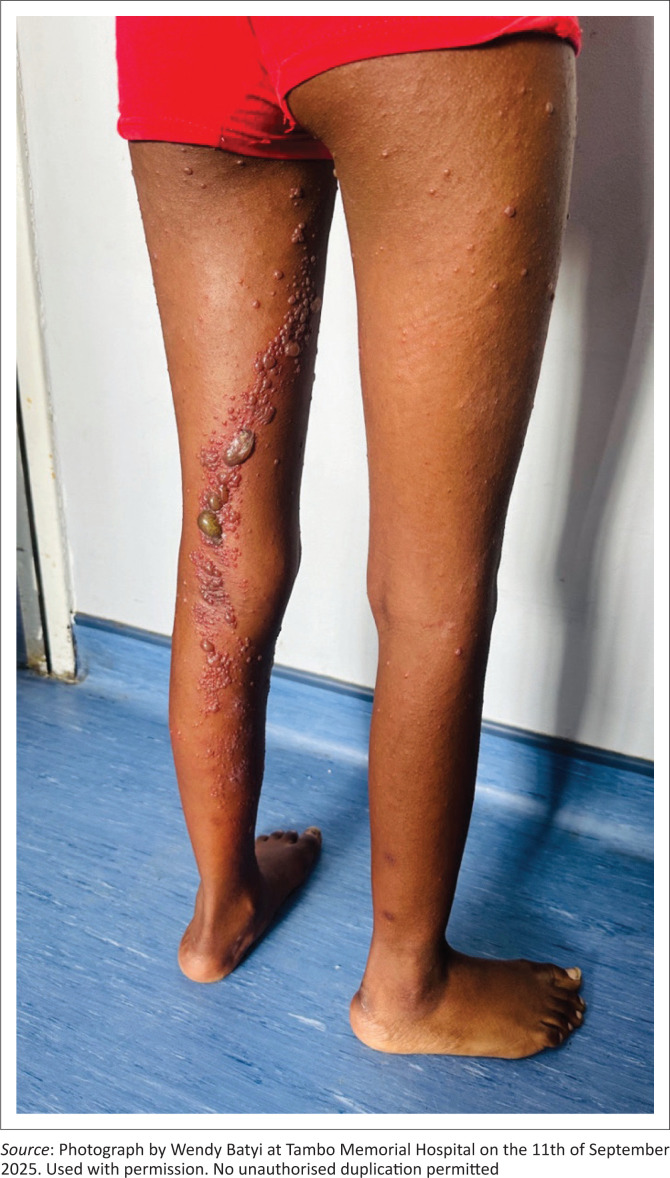
Zoster. Distributed along S1–S2 dermatomes.

An 11-year- old girl presented with a 5-day history of skin lesions. Her initial symptoms were a fever and pain in her left hip, which limited her ability to walk. After 2 days, scattered papular and vesicular lesions appeared on her left hip, spreading to the lower trunk, upper limbs, face and then the rest of the lower limbs (Figure 1 and Figure 2). When the lesions spread to both lower limbs, that is when blisters in a linear pattern were observed on the posteromedial aspect of the left lower limb.

**FIGURE 3:**
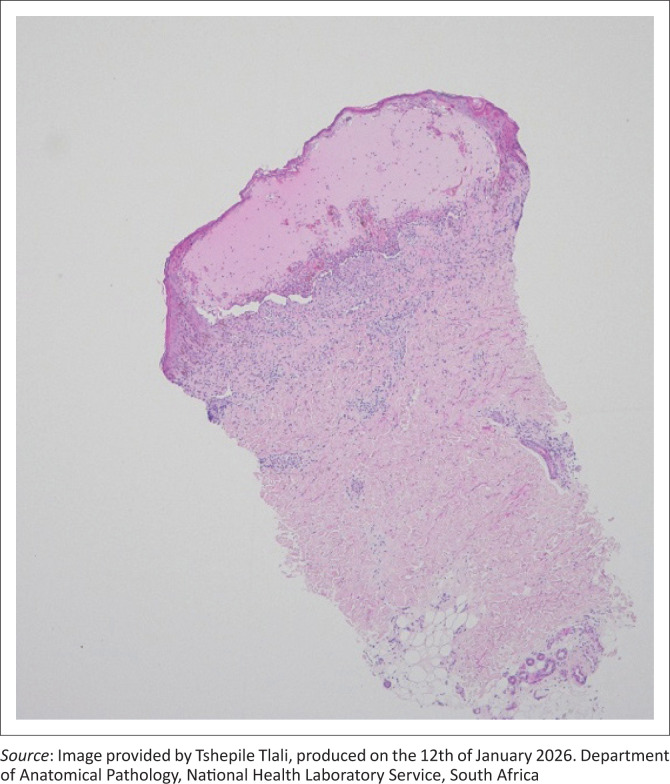
Low-power micrograph of skin punch biopsy showing blister.

No previous history of varicella infection or vaccination could be elicited. She did not have any close contacts infected with varicella. Her mother was known to be HIV-positive, and the patient received nevirapine and zidovudine postnatally. Her birth and 10-week HIV polymerase chain reaction (PCR) were negative. She was breast-fed for 18 months but was not retested following cessation of breastfeeding. On presentation, she had generalised vesicular lesions and linear left leg vesicular lesions (S1–S2 dermatomes). She did not have mucosal involvement. Initial investigations included an HIV ELISA, which was reactive. A full blood count, urea and electrolytes, C-reactive protein, blood culture, urine microscopy, culture and sensitivity were done because the patient was suspected of having sepsis, considering the accompanying fever and tachypnoea she had. Subsequent testing revealed an absolute cluster of differentiation 4 (CD4) count = 578/µL and an HIV viral load = 19 287 copies/mL. Cerebrospinal fluid analysis was positive for Varicella-Zoster virus and Epstein- Barr virus. A skin swab cultured no pathogenic bacteria (see Table 1). Chest and left leg X-rays were normal. Skin biopsy of a leg and trunk lesion revealed histological features of VZV infection with an intra- epidermal blister containing serous fluid and neutrophils (Figure 3).

**TABLE 1:** Biochemical profile.

Variable	Results on admission	Results during admission	Reference values
White cell count	5.94 × 10^9^/L	6.70 × 10^9^/L	3.92 – 10.20 × 10^9^/L
Haemoglobin	12.1 g/dL	10.6 g/dL	10.3 g/dL – 15.5 g/dL
Platelet count	141 × 10^9^/L	160 × 10^9^/L	180 10^9^/L – 440 × 10^9^/L
Sodium	121 mmol/L	129 mmol/L	136 mmol/L – 145 mmol/L
Potassium	4.5 mmol/L	4.5 mmol/L	3.4 mmol/L – 4.7 mmol/L
Urea	4.0 mmol/L	7.3 mmol/L	2.1 mmol/L – 7.1 mmol/L
Creatinine	51 μmol/L	77 μmol/L	37 μmol/L – 63 μmol/L
Calcium	2.06 mmol/L	2.15 mmol/L	2.15 mmol/L – 2.64 mmol/L
Magnesium	0.94 mmol/L	0.99 mmol/L	0.66 mmol/L – 0.91 mmol/L
Phosphate	1.23 mmol/L	1.02 mmol/L	1.05 mmol/L – 1.70 mmol/L
CRP	20 mg/L	-	< 10 mg/L
HIV Elisa	Reactive	-	-
Absolute CD4	578 cells/μL	-	650 cells/μL – 1500 cells/μL
Urine MC&S	-	No growth after 1 day	-
Skin swab	-	No growth after 2 days	-
Blood culture	-	No growth after 5 days	-
CSF	-	Epstein-Barr virus positive Varicella-zoster virus positive	-

CRP, C- reactive protein; MC&S, Microscopy, culture & sensitivity; CSF, Cerebrospinal fluid.

**FIGURE 4:**
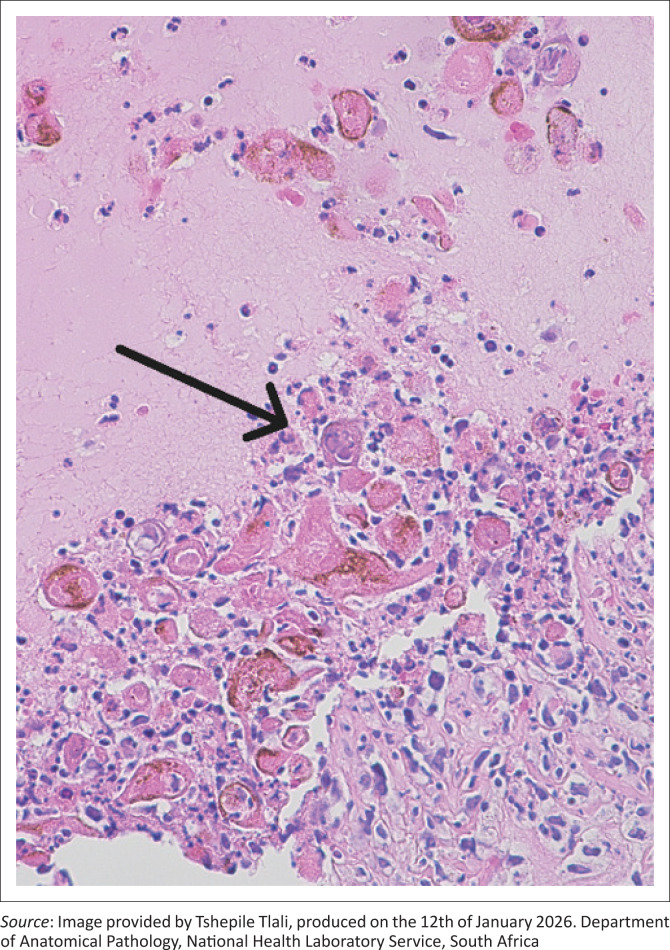
High-power micrograph of skin punch biopsy showing viral inclusion.

Adjacent keratinocytes demonstrated eosinophilic intranuclear inclusions with chromatic margination, nuclear moulding and multinucleation (Figure 4, arrow). Immunochemistry was positive for varicella-zoster virus and negative for the herpes simplex virus (images not shown). She received 400 mg of intravenous acyclovir 8-hourly. Ampicillin and gentamicin were started for suspected sepsis but were discontinued after 2 days.

Subsequently amoxicillin-clavulanic acid was given for 5 days while waiting for blood culture results. Carbamazepine (200 mg) was initially prescribed for neuropathic pain but was replaced with amitriptyline (10 mg) after 1 day, as this is the recommended drug for herpetic neuralgia. The patient received acyclovir for 7 days and was then discharged with healing skin lesions to start antiretroviral treatment as an outpatient.

## Discussion

Zoster is less common in children than in adults, and risk factors include primary VZV infection in infancy and immunosuppressive state, e.g. chemotherapy, other immunosuppressive drugs and HIV infection.^1,4,7^ Zoster typically occurs in adults, especially the elderly, as a result of a decline in immunity or immunocompromise. Consequently, its occurrence in children is not as common as in adults but does increase with age.

Two immunocompetent children with simultaneous varicella and zoster have been reported.^1,6^ It is not clear why these children had such a short latency period with rapid VZV reactivation. A possible explanation is that viraemia during primary infection may cause immediate replication in the ganglia.^1^ In our patient, untreated HIV was the only identified risk factor. Considering our patient’s immunosuppressive state, a diagnosis of disseminated zoster cannot be excluded. She may have had prior asymptomatic, mildly symptomatic or forgotten varicella. What also supports this is that her presentation of left hip pain is in keeping with prodromal pain before the cutaneous manifestations of zoster. As described previously, the patient presented with scattered vesicles of the left hip before the emergence of the blisters in the dermatomes, but this was according to history, and the evolution was not observed by a clinician. Therefore, the history from the mother may have been distorted, and the sequence of evolution of the disease may not have been presented correctly.

## Conclusion

Though rare, contemporaneous varicella and zoster occur in children. It can affect immunocompetent and immunocompromised children. Disseminated zoster is not uncommon in HIV-positive children who are not on antiretrovirals. Clinical vigilance is especially important for the immunocompromised child, such as in our newly diagnosed HIV patient, so as to institute prompt treatment with acyclovir, as the risk of complications is higher in this group of patients.
